# Genotype and environment factors driven licorice growth and rhizospheric soil fungal community changes

**DOI:** 10.3389/fmicb.2023.1308412

**Published:** 2023-11-23

**Authors:** Tingting Han, Xianen Li, Dan Luo, Changhao Ji, Caixia Chen, Chao He

**Affiliations:** ^1^Institute of Medicinal Plant Development, Chinese Academy of Medical Sciences and Peking Union Medical College, Beijing, China; ^2^College of Life Sciences, Hebei University, Baoding, China

**Keywords:** licorice, genotype, environment, active ingredient, soil physicochemical properties, rhizospheric soil fungal community

## Abstract

**Introduction:**

Licorice (*Glycyrrhiza uralensis* Fisch.) is a widely recognized significant form of medicine in China, with a long-standing history and extensive usage. It is considered the oldest and most prevalent herbal medicine in China. Currently, the licorice market is confronted with the primary challenges of mixed genotypes, inconsistent quality, and inadequate glycyrrhizic acid content.

**Methods:**

We conducted field experiments to investigate the impact of various cultivation locations on the growth characteristics, active ingredients, rhizospheric soil physicochemical properties and fungal communities of licorice that ten different genotypes.

**Results:**

The findings indicated significant variations in these parameters across ten different genotypes of licorice originating from two distinct production regions. The growth characteristics of licorice were primarily influenced by genotype, whereas the active ingredients of licorice were mainly influenced by environmental factors and soil physicochemical properties. Furthermore, the rhizospheric soil physicochemical properties of licorice plants were more influenced by environmental factors than genotypes. Additionally, the distribution of rhizospheric soil fungi in licorice plants of the same genotype exhibited significant variations across different cultivation areas. The utilization of structural equation model synthesis reveals variations in the quantity and strength of pathways that influence the growth characteristics, active ingredients, and rhizospheric soil microbial community of licorice across different cultivation regions.

**Discussion:**

Based on the main results, according to its growth characteristics and active ingredients, Z009 proved to be the most suitable genotype for cultivation in Jingtai. From a perspective centered on the active ingredient, Z010 proved to be the most optimal genotype for licorice cultivation in both production areas. Our study aims to enhance the understanding of the ecological adaptability of various genotypes of licorice resources and to identify appropriate licorice genotypes for specific cultivation regions. This research holds significant practical implications for enhancing the yield and quality of licorice, thereby improving its overall development.

## Introduction

Licorice (*Glycyrrhiza uralensis* Fisch.) is a perennial leguminous herb. Its roots and rhizomes are widely used in traditional Chinese medicine prescriptions, and can be used for clearing heat and detoxicating, relieving cough and phlegm, tonifying the spleen and stomach, and reconciling various medicines (Zhu et al., 2016; Zhao et al., 2017; Bao et al., 2018). In China, it is mainly found in arid, semi-arid or desert areas such as Xinjiang, Inner Mongolia, and Gansu. Licorice is one of the most commonly used herbs with the same origin as food, ranking the top three in respect of herbs appearing in traditional Chinese medicine prescriptions, and is also widely applied in the manufacturing of food, health products, cosmetics, etc. (Zhao et al., 2010; Carocho et al., 2015; Ram, 2015). The cultivation area of licorice spans more than 300 million square meters in China, and the annual consumption exceeds 60,000 tons. Licorice also plays an important role as a windbreaking and sand-fixing plant in China’s northern arid and semi-arid regions, which is of great importance for inhibiting further desertification in arid regions of Northwest China (Liu et al., 2007; Zhang J. T. et al., 2011; Li et al., 2016). However, with the development of the modern pharmaceutical industry and the comprehensive exploration of licorice resources, wild licorice resources in China have been over-used, and the ecological environment in some areas has been seriously damaged. Therefore, more and more attention has been paid to the standardized artificial cultivation of licorice. Most of the seeds come from wild genotype with rich variation (Li et al., 2019; He et al., 2020). In addition, licorice plants have a certain similarity in morphology, so they are easily confused when collecting seeds. This leads to in uneven quality of cultivated licorice products on the market, greatly affecting the healthy development of the licorice industry. Therefore, screening high-quality licorice genotype resources is not only the primary prerequisite to ensuring the quality of licorice, but also can further promote the identification and protection of genotype resources, paving the way for the cultivation of new varieties.

In the process of growth and accumulation of medicinal components, medicinal plants are subject to the dual stress from internal genetic factors and external environmental changes (Yang, 2016). Genetic factors, as the basis for the sustainable development of Chinese herbal medicine resources, are particularly important in evaluating the authenticity of Chinese herbal medicines and breeding high-quality varieties. Among them, the random amplified polymorphic DNA technique has been a widely used as molecular marker for authentic identification of multiple medicinal plants (Wang et al., 2003; Li and Li, 2006; Cao et al., 2010). There are also many research reports on the use of functional genes of medicinal materials. For example, one of the reasons for the formation of licorice authenticity may be the single nucleotide polymorphism on β-AS gene 94 of licorice from different origins (Xi et al., 2013; Zang et al., 2015). Using trnh-psbA DNA barcodes to verify the authenticity of dried tangerine peel, results showed that there were significant differences between the trnh-psbA DNA sequence and orange peel, providing preliminary evidence at the molecular level for the identification of the authenticity of tangerine peel (Yang and Chen, 2015). Compared with intrinsic genetic factors, the role of extrinsic environmental factors on the growth and development of medicinal plants cannot be ignored, such as geographic climate, natural selection, and human interference (Abdala-Roberts et al., 2016; Ding et al., 2022). Appropriate high temperature conditions can promote the accumulation of camptothecin content and its yield per plant (Liang et al., 2016), and drought stress can significantly promote the synthesis of tanshinones in hairy roots of *Salvia miltiorrhiza*, and produce salvianolic acid B, rosmarinic acid, caffeic acid, and 3,4-trihydroxybenzenepropanoic acid in *S. miltiorrhiza* leaves (Sheng and Chen, 2013; Liu et al., 2015). Studies have reported that *Panax notoginseng* grows well at a light transmittance of about 10%. In low light conditions, *P. notoginseng* mainly adopts a conservative strategy for slow carbon acquisition and carbon consumption (Shuang et al., 2022). A large number of studies show that the quality of Chinese herbal medicines with the same origin can be significantly different in different production areas, and is closely related to the climate and soil conditions (Lu et al., 2006). Even for the same plant, different environmental stresses can induce the formation and accumulation of different secondary metabolites, and specific environmental stresses promote the accumulation of specific secondary metabolites in plants (Wang, 2019). *S. miltiorrhiza* plants from 12 different production areas were observed. The content of tanshinone IIA was significantly different in *S. miltiorrhiza*, among which the content of tanshinone IIA in salvia plants from Anhui was significantly lower than that in other production areas (Liang et al., 2020). When verbena plants of different origins are subject to the same gradient of light, temperature, and pH, the growth and physiological indicators show different trends (Javaid et al., 2018). At present, there are many reports on the relationship between different ecological factors and the yield and quality of medicinal plants, but most of them focus on the effects of a single type of ecological factor on the yield and secondary metabolites of medicinal plants. However, there are no systematic research reports available on the internal genetic factors and external environment or the quality of medicinal plants.

The plant rhizospheric is the area where the root’s own life activities and metabolism have the most direct and biggest impact on the soil, and it is also the place where the root system interacts with the soil and microorganisms (Butler et al., 2003; Garcia and Kao-Kniffin, 2018). Rhizospheric microorganisms are important part of plant rhizospheric soil and the most active members in daily metabolic activities. They secrete and release various soil enzymes and participate in biochemical processes such as organic matter degradation, humus synthesis, and nutrient cycling. The assembly of plant rhizospheric microbial communities are controlled by the complex interactions among microorganisms, plant hosts, and the environment factors. The microbial communities are in dynamic fluctuation and interact with each other, thus forming a complex plant rhizospheric microbial network. Studies have shown that the soil physicochemical properties in different regions macroscopically affect the microbial communities in the soils of that region, while factors such as plant species and growth stages determine which microorganisms can become enriched in the rhizospheric (Walters et al., 2018). The relationship between host plants and rhizospheric microorganisms is intricate, ranging from mutually beneficial symbiosis to antagonistic inhibition, depending on the species of plants or hosts and different environmental factors at a specific time. The difference of microbial community structure in plant rhizospheric soils and non-rhizospheric soils is largely attributable to strong plant selection or inhibition of microbial communities (Bulgarelli et al., 2015; Tian et al., 2020). Studies on the growth of sorghum and the plant rhizospheric microbiome under drought stress have shown that sorghum has a strong screening effect on the soil fungal species library (Gao et al., 2020). Rhizospheric microorganisms can affect plant growth and stress resistance in both direct and indirect ways. Mycorrhizal fungi can form symbiotic mycorrhizae with most terrestrial higher plants, increase the contact area with the soil through the mycelial network of the roots, and enhance the absorption of nitrogen and phosphorus by medicinal plants (Davison et al., 2015). Mycorrhizal fungi can significantly increase the content of nitrogen, phosphorus, potassium, and other nutrients in the rhizospheric soil of *Paris polyphylla* var., and enhance the enrichment of Mg, Na, Zn, and Ni, which is conducive to its growth and quality formation (Zhang et al., 2019). Egamberdieva et al., have found that co-inoculation with *Mesorhizobium* sp. NWXJ19 and *Pseudomonas extremorien* talis TSAU20 significantly improved the salt tolerance of licorice plants and increases the yield and number of nodules of licorice (Egamberdieva et al., 2016). The application of *Bacillus subtilis* enhances the expression of genes related to drought resistance and salt tolerance in *Arabidopsis* (Woo et al., 2020). Rhizospheric soil fungi are one of the indicators of soil ecosystem health, because their genetic and functional diversity plays an indispensable role in the “plant-soil-microbe” interaction system. The composition of the fungal community in rhizospheric soil is known to be extensive and diverse, and it plays a vital role in the decomposition of organic matter and the cycling of nutrients in soil (Mendes et al., 2013). The topic at hand is intricately linked to the growth and metabolism of plants, making it a significant biological factor that influences the production of active compounds in medicinal plants. Simultaneously, as plants grow, there is a gradual alteration in rhizospheric metabolic activities, particularly in the release of certain organic compounds. These changes have a significant impact on the composition and distribution of fungi in the rhizospheric soil (Raaijmakers et al., 2009; Shi et al., 2011). It’s also bringing us another new perspective to study and quantify environmental and genotype factors for plant growth and accumulation of active ingredients.

In this study, different licorice genotypes collected from distinct main licorice production areas were planted in two locations under different environmental conditions, and consistent field management methods were adopted in order to elucidate the effects of various genotype and ecological environmental factors on licorice quality formation and root development. The impact of changes in the fungal community in different regions was investigated, and the following questions were responded: (1) What are the main ecological factors that affect the growth and accumulation of active ingredients of licorice in different production areas? (2) How does the composition of the fungal community in licorice rhizosphere change in different production areas? (3) How are the main factors affecting the formation of licorice quality transmitted? How can the effect be quantified? Studying these problems will help to reveal the law of licorice growth, accumulation of active ingredients, the changes of fungal community in rhizospheric soil, provide a theoretical basis for standardized cultivation of licorice in different production areas and regulation of accumulation of active ingredients in the future.

## Materials and methods

### Field experiment design and sample collection

In April 2020, we conducted experimental arrangements at licorice planting bases in Jingtai County, Baiyin City, Gansu Province, and Chifeng City, Inner Mongolia Autonomous Region, China (Table 1). The climate data of the current year is obtained by measuring the meteorological stations installed in the local. The annual average temperature in Jingtai County was 8.2°C, and the annual average rainfall was 185.3 mm. In the experimental field, in terms of basic soil nutrients, there were 11.06 mg/g of organic matter, 46.15 mg/kg of available nitrogen (N), 5.78 mg/kg of available phosphorus (P), 124.72 mg/kg of available potassium (K), and the soil pH value was 7.89. The annual average temperature in Chifeng was 7.5°C, and the annual average rainfall was 400 mm. In the experimental field, in terms of basic soil nutrients, there was 13.77 mg/g of organic matter, 62.07 mg/kg of available nitrogen (N), 13.22 mg/kg of available phosphorus (P), 145.56 mg/kg of available potassium (K), and the soil pH value was 7.17.

**Table 1:** Parameters of climatic conditions at different sampling sites.

Sampling locations	Average temperature (°C)	Mean rainfall (mm)	Annual sunshine hours (h)	Relative humidity (%)
CF	7.5 ± 0.18	400.9 ± 7.21	2,846 ± 8.94	47.2 ± 1.32
JT	8.2 ± 0.13	185.3 ± 5.87	2,726 ± 6.62	40.8 ± 1.55

The materials used in this study are 10 genotypes (Z001, Z002, Z003, Z004, Z005, Z006, Z007, Z008, Z009, and Z010) collected by the research team from the main licorice production areas of Inner Mongolia, Xinjiang, and Gansu, as shown in Table 2. We planted these 10 different genotypes of licorice in two standardized licorice cultivation bases in Chifeng, Inner Mongolia and Jingtai, Gansu, with consistent field management methods. Such as a unified application of base fertilizer before planting, unified watering after sowing, the main natural precipitation to replenish water after the seeds grow into seedlings, and artificial weeding when weeds are strong (He et al., 2019a). Specific field experiment design is as follows: The experimental plot for each genotype covers an area of 6 m^2^ (3 m × 2 m). Four rows of licorice were planted in each plot, with a spacing of 30 cm × 15 cm between plants and between rows. A 20 cm drainage ditch was set between plots. Five replicates were set up for each treatment. On November 10, 2021, licorice plant samples were collected using the method of random mixed sampling at multiple points in each plot. Four healthy plant samples were collected from each plot, and a total of 20 plants were collected for each treatment. In this study, the cultivation time of licorice was 2 years.

**Table 2:** Genotype sources of different genotypes of licorice.

Genotype number	Genotype source
Z001	Bohu County, Bayingol Mongolian Autonomous Prefecture, Xinjiang Uygur Autonomous Region (39°24′N, 85°38′E)
Z002	Yanchi County, Wuzhong City, Ningxia Hui Autonomous Region (37°11′N, 107°26′E)
Z003	Yining City, Xinjiang Uygur Autonomous Region (43°52′N, 80°43′E)
Z004	Korla City, Xinjiang Uygur Autonomous Region (41°18′N, 42°15′E)
Z005	Altay City, Xinjiang Uygur Autonomous Region (47°22′N, 88°37′E)
Z006	Bayannur City, Inner Mongolia Autonomous Region (41°15′N, 107°22′E)
Z007	Hangjin Banner, Ordos City, Inner Mongolia Autonomous Region (39°16′N, 107°31′E)
Z008	Minqin County, Wuwei City, Gansu Province (38°12′N, 102°28′E)
Z009	Huining County, Baiyin City, Gansu Province (36°24′N, 104°11′E)
Z010	Changji City, Xinjiang Uygur Autonomous Region (44°19′N, 86°28′E)

Following this, the licorice sample was removed intact from the sampling point, the soil attached to the roots of the plant was gently shaken off as a rhizospheric soil sample. Plant and soil samples were collected separately (0–30 cm soil layer). All samples of licorice were packed and numbered according to different genotypes of different plots as taxonomic units. The plant samples were used to analyze the growth characteristics and active ingredients of licorice. The rhizospheric soil samples of plants from different treatment groups were sieved (<2 mm mesh) and divided into two fractions. A fraction was placed at 25°C for air drying to determine soil physicochemical properties, and the other fraction was placed in a refrigerator at −80°C for analyzing the diversity of rhizospheric soil fungal communities.

### Growth parameters of licorice

The root system of licorice plants with various genotypes harvested in different production areas was gently washed with water to remove the attached sandy soil. The aboveground and underground parts of 20 healthy licorice plants were separated and the root fresh weight was measured. Each individual root system was then floated in a plexiglass tray in water with approximate 1 cm depth, and a scanner (EPSON perfect V800 Photo, Japan) was used. Root morphological characteristics, including total root length, mean root diameter, and root branch number, were assessed using the Win-RHIZO image analysis system (Regent Instruments, Quebec, QC, Canada) (He et al., 2019a). Finally, the root samples of fresh licorice plants were dried at 70°C and reserved for the detection of active ingredients.

### Active ingredients of licorice

High Performance Liquid Chromatography (HPLC) was used to determine the content of flavonoids in licorice (He et al., 2021a,b). Samples and standards were prepared in accordance with the guiding principles of the Pharmacopeia of the People’s Republic of China (2020 Edition). Glycyrrhizic acid, liquiritin, liquiritigenin, and isoliquiritigenin were purchased from the National Institutes for Food and Drug Control (NIFDC) in Beijing with purity greater than 98%. Among them, glycyrrhizic acid and liquiritin are the indicator ingredients specified in the Pharmacopeia of the People’s Republic of China. Additionally, liquiritigenin and isoliquiritigenin are common and detectable active ingredients with obvious pharmacological activity, while the content of other active ingredients is low and difficult to reach the detection limit. The detection method is internal standard method.

The working parameters are as follows: the chromatographic column is Xselect (R) HSS T3 C18 column (250 × 4.6 mm, 5 μm. Waters Corporation); the mobile phase is acetonitrile (Phase A)—phosphate aqueous solution (0.5%) (Phase B). The separation is conducted in the gradient elution mode (Supplementary Table S1) at 30°C with a flow rate of 1 mL/min; dual wavelength detection, λ1 is 237 nm, λ2 is 365 nm; the injection volume is 10 μL; the retention time of each active ingredient is as follows: Glycyrrhizic acid: 37.028 min, Liquiritin: 9.054 min, Liquiritigenin: 23.850 min, Isoliquiritigenin: 35.109 min.

### Physicochemical properties of licorice rhizospheric soil

The rhizospheric soil samples closely adhered to the roots were collected, screened with a 2 mm sieve, and dried at room temperature. Each dried soil sample (0.2 g) was digested in a 10:1:2 mixture composed of 10 mL perchloric acid (12.7 mol/L), sulfuric acid (18 mol/L) and water using a Mars 6 microwave digestion system (CEM, Matthews, NC, United States) until a transparent liquid was obtained (He et al., 2019b). Soil organic matter was determined as the percentage of organic carbon oxidized by dichromate in H_2_SO_4_ (Rowell, 1994; Chen et al., 2021). The contents of available nitrogen (N) and available phosphorus (P) was determined by using alkaline hydrolysis diffusion (Rowell, 1994; Chen et al., 2021) and the chlorostannous-reduced molybdophosphoric blue method (Olsen et al., 1954; Chen et al., 2021) respectively. The content of available potassium (K) was determined by Flame Photometer (Jackson, 1973; Chen et al., 2021).

### High-throughput sequencing analysis of rhizospheric soil fungal communities

The Powersoil^®^DNA Extraction Kit (Mo Bio, Carlsbad, United States) was used to extract 0.25 g of total genomic DNA from rhizospheric soil samples. Extracted DNA quality was assessed by using 0.1% (w/v) agarose gel electrophoresis, and purification and concentration of DNA were determined using a NanoDropTM 1,000 spectrophotometer (Thermo Fisher Scientific Inc., USA). The universal primer ITS1F (5′-CTTGGTCATTTAGGAAGTAA-3′)-ITS2R (5′-GCTGCGTTCTTCATCATGATGC-3′) was used to study the microbial community through the internal transcribed fragment (ITS) region of the target fungi. Three parallel tests with polymerase chain reaction (PCR) were performed using a 20 μL mixture consisting of 4 μL L5 × FastPfu buffer (16 s v3-v4)/2 μL 10× buffer (ITS), 2 μL LdNTP, 0.8 μL forward and reverse primers each, and 1 μL the other two primers each, 0.4 μL FastPfu polymerase (16S v3-v4)/0.2 μLrTaq polymerase (ITS), 0.2 μLBSA and 10 ng template DNA (He et al., 2021b). PCR amplification conditions for each primer were designed using the following thermal program: initial denaturation at 95°C for 3 min, 28 cycles of denaturation at 95°C for 30 s, annealing at 55°C for 30 s, extension continued at 72°C for 45 s, and a final extension at 72°C for 10 min. PCR products were detected by gel electrophoresis (2% agarose) and further purified with the AxyPrep™DNA Gel Extraction Kit (Axygen BioSciences Inc., United States). QuantiFluor^™^dsDNA system equipped with QuantiFluor^™^- was used for quantitative ST fluorometer and PCR tube adapter (Promega, United States). Samples were sequenced on an Illumina MiSeq PE 300 platform using the paired end option [2 × 300 base pairs (bp)] on the Beijing Norohezhiyuan Environmental Genome platform.

### Bioinformatics analysis

In this study, original fastq files were demultiplexed, quality filtered and merged by Trimmomatic and short-read fast length adjustment (FLASH, Johns Hopkins University, USA). Sequences with length <50 bp, average quality score <20, and base ambiguity were removed. Based on the overlap between PE reads, sequences with an overlap time of more than 10 bp were merged. Base mismatch sequences were removed during primer matching. Data analysis was performed using data processing software provided by Shanghai Mingzhi Biomedical Technology Co., LTD. Based on the Usearch software platform, the obtained high-quality sequences were clustered by operational taxonomic units (OTU) with 97% similarity. OTU representative sequences with 97% similarity levels were classified using the ribosomal database project (RDP) classifier Bayesian algorithm and compared to the Fungus (ITS) federated database with a confidence threshold of 0.7. Then RDP was used to collate the functional gene database from genebank (release7.3^1^), to obtain species annotation information. All samples were subsampled according to the minimum number of sample sequences. According to the OTU clustering and annotation results, R language tools were used for the dilution curve, Venn diagram, and community composition analysis, and the alpha diversity index was calculated with mothur version 1.30.2 analysis software. Other data analysis and mapping were performed using SPSS 22.0 and origin 9.0 software.

### Statistical analysis

All statistical analyses were performed in SPSS 21.0 (SPSS Inc., Chicago, IL, United States). One-way analysis of variance (ANOVA) was used to analyze the root biomass, total root length, root diameter, and root branch number of different licorice genotypes in different production areas.

Two-way ANOVA was used to investigate the effects of genotype, environmental factors, and genotype × environmental factors on growth characteristics, active ingredients, and soil physicochemical parameters. The two-way ANOVA was analyzed by using the Duncan multiple range test, where *p* < 0.05, and was considered to be statistically significant (He et al., 2019b). The values in the figure were the average of at least three replicates. At the same time, the R-3.5.3 vegan program package was used to carry out non-metric multidimensional scaling analysis (NMDS) and plot the soil fungal and bacterial community composition data.

The R-3.5.3 vegan package Adonis command was used to conduct multivariate analysis of variance (PerMANOVA) on the variation of soil fungal community composition. Fungal OTU dilution curves in each treatment were calculated using the specaccum function in the vegan package (Oksanen et al., 2016). According to the studies, the preference analysis of environmental factors (water, soil organic matter, available nitrogen, available phosphorus, available potassium)/microorganisms was carried out (Huang et al., 2018; Yao et al., 2019). The visualization analysis of soil microbial network in different treatments was carried out with CYTOSCAP 3.4.0.

FUNGuild^2^ database software was used to forecast and analyze the fungus ecological function and define the nutrition type and nutrition level, with different uncertainty (may, might, and most likely). The fungal community whose nutrient type was compound nutrient type was included in “other fungi,” and those identified as compound multifunctional type were classified as “other pathogens/saprophytic fungi” under nutrient type. The genera with abundance greater than 200 at OTU classification level was used to construct the visual analysis map of soil bacterial network in different treatments.

In order to better understand beneficial, pathogenic, and neutral fungi and bacteria, the significance of the richness of important microbial communities in different treatments was analyzed by network model. We evaluated the effects of DSE, water, and their interactions on licorice biomass, microbial community composition, and fungal OTU and bacterial OTU diversity using two-way ANOVA (He et al., 2019a).

The studies showed significant differences in genotype, thus environmental parameters (average temperature, mean rainfall, annual sunshine hours, and relative humidity), soil parameters (organic matter, available N, P, and K), growth parameters (root biomass, total root length, root diameter, and root branch number), active ingredients (glycyrrhizic acid, liquiritin, liquiritigenin, and isoliquiritigenin), and fungal abundance were analyzed by preference Heatmap (Huang et al., 2018; Yao et al., 2019).

We also used the variational variance decomposition method to evaluate the effects of different genotypes, environmental parameters, and soil parameters on plant biological traits, active ingredients, and soil fungal abundance of licorice from different habitats (He et al., 2021a). At the same time, R-3.2.2 package ecological software (Goslee and Urban, 2007) and Amos 21.0 (Maximum Likelihood) were further used to test the effects of genotype, environmental factors, and soil factors on the growth traits, active ingredients, and soil fungal communities of licorice by Mantel test and SEM, in an effort to clarify the action and intensity of the three factors on the formation of licorice quality (He et al., 2021b).

### Accession numbers

The Illumina MiSeq sequence datasets are available at the NCBI Sequence Read Archive BioProject ID PRJNA1016103 (SUB13828680).

## Results

### Plant growth parameters

**Figure 1:**
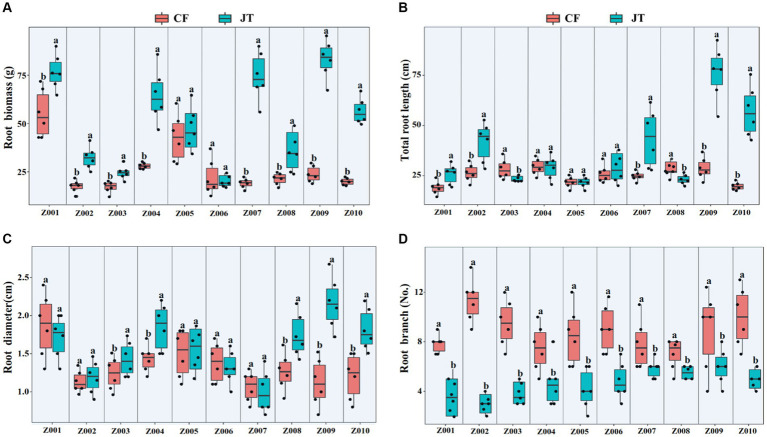
Plant growth parameters of different genotype licorice in different cultivation locations: **(A)** Root biomass, **(B)** Total root length, **(C)** Root diameter, and **(D)** Root branch. CF, Chifeng; JT, Jingtai. The different letters above the error line indicate significant difference at *p* < 0.05.

The effects of different genotype and environmental factors on the growth parameters of licorice were significantly different (Figure 1). In terms of root biomass, the licorice in Jingtai was significantly higher than Chifeng, especially Z001, Z004, Z007, Z009, and Z010, which had significantly higher biomass than Chifeng when they were grown in Jingtai. In Chifeng the biomass of Z001 and Z005 was significantly higher than other genotypes (Figure 1A). In terms of total root length, the biomass of Z002, Z007, Z009, and Z010 grown in Jingtai was significantly higher than that in Chifeng, and the biomass of genotypes Z009 was the highest (Figure 1B). In terms of root diameter, the root diameters of Z003, Z004, Z008, Z009, and Z010 grown in Jingtai were significantly higher than those in Chifeng. There were no significant differences among other genotypes of different origins (Figure 1C). In terms of root branch number, all genotypes grown in Chifeng had significantly more root branches compared with those in Jingtai (Figure 1D). Through two-way ANOVA, we found that genotype and environmental factors of different origins had significant interaction effects on root biomass and root branch number, and environmental factors had extremely significant effects on root biomass and root branch number (Table 3).

**Table 3:** Two-way ANOVA of the effect of genotype and environment condition on plant growth parameter of licorice plants.

	Root biomass (g)		Total root length (cm)		Root diameter (mm)		Root branch (No.)	
	*F*	*P*	*F*	*P*	*F*	*P*	*F*	*P*
Genotype	6.3	0.032	3.6	NS	7.8	0.013	3.8	NS
Environment	11.5	<0.001	5.1	0.046	2.0	NS	10.2	<0.001
Genotype × Environment	7.0	0.024	1.9	NS	4.5	NS	6.1	0.030

### Contents of active ingredients

**Figure 2:**
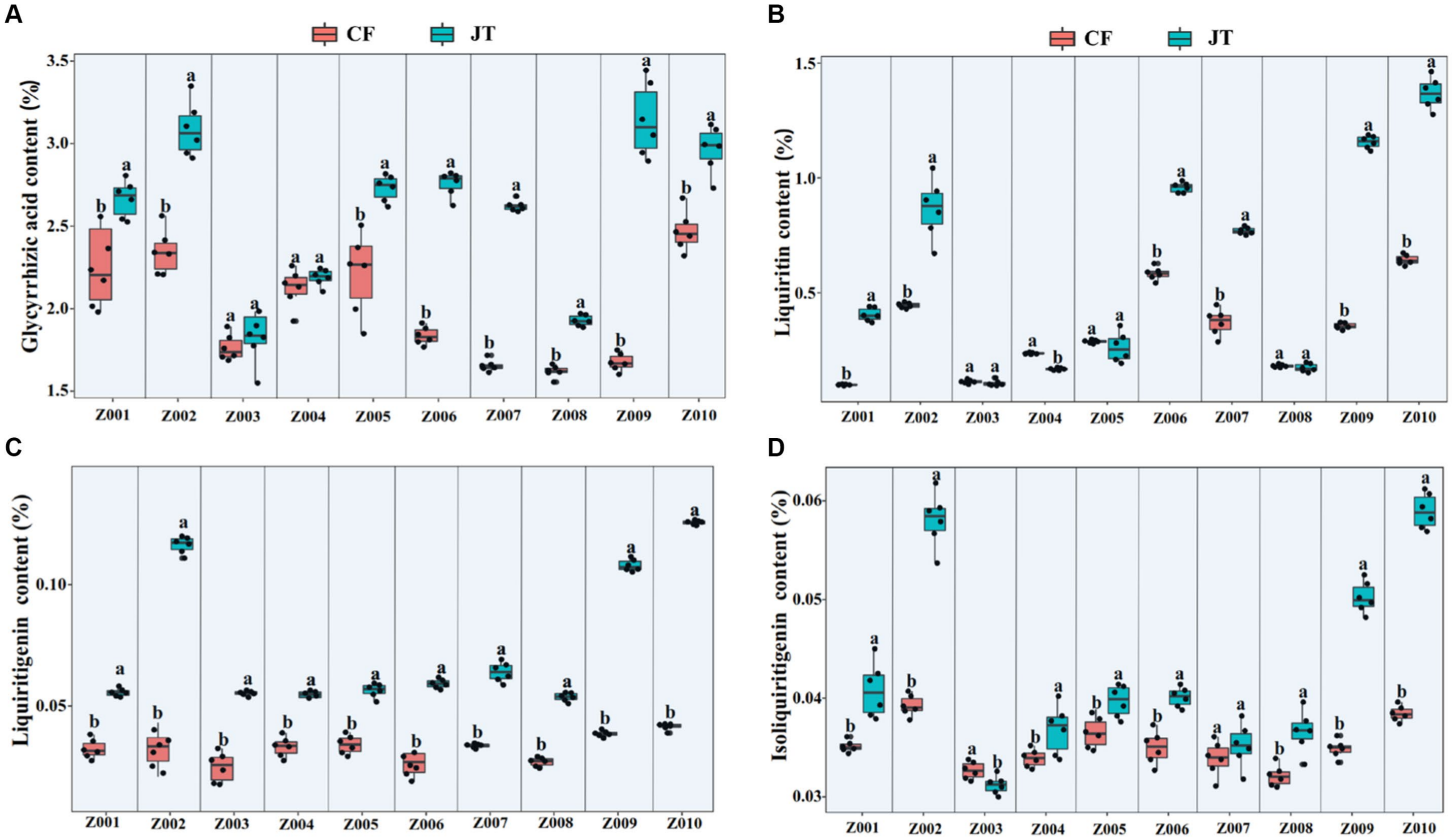
Active ingredient contents of different genotype licorice in different cultivation locations: **(A)** Glycyrrhizic acid content, **(B)** Liquiritin content, **(C)** Liquiritigenin content, and **(D)** Isoliquiritigenin content. CF, Chifeng; JT, Jingtai. The different letters above the error line indicate significant difference at *p* < 0.05.

According to HPLC determination of the content of glycyrrhizic acid, liquiritin, liquiritigenin and isoliquiritigenin in licorice, it was found that the effects of different genotype and environmental factors on the active ingredients parameters of licorice were significantly different (Figure 2). In terms of glycyrrhizic acid content, except for Z003 and Z004, the glycyrrhizic acid contents of other licorice genotypes in Jingtai were higher than those in Chifeng, while the glycyrrhizic acid content of Z002, Z009, and Z010 were significantly higher than that of another genotype (Figure 2A). In terms of liquiritin content, except for Z003, Z004, Z005, and Z008, the liquiritin content of another genotype was higher than that in Chifeng. It is worth mentioning that the content of Z004 in Jingtai was lower than that in Chifeng (Figure 2B). In terms of liquiritigenin content, the content of liquiritigenin in each variety of licorice in Chifeng was not significantly different, but the difference in different varieties of licorice in Jingtai was significant, among which Z002, Z009, and Z010 were significantly higher than those in other treatments (Figure 2C). In terms of isoliquiritigenin content, except for Z003 and Z007, the content of isoliquiritigenin grown in Jingtai was significantly higher than that of Chifeng, and the content of Z002, Z009, and Z010 was higher than that of other varieties. However, there was no significant difference in the content of isoliquiritigenin among different varieties in Chifeng (Figure 2D). Through two-way ANOVA, we found that genotype and environmental factors of different origins had significant interaction effects on the contents of glycyrrhizic acid, liquiritin, and liquiritigenin, and genotypes had extremely significant effects on liquiritin, and environmental factors also had extremely significant effects on isoliquiritigenin (Table 4).

**Table 4:** Two-way ANOVA of the effect of genotype and environment condition on plant physiological parameter of licorice plants.

	Glycyrrhizic acid (%)		Liquiritin (%)		Liquiritigenin (%)		Isoliquiritigenin (%)	
	*F*	*P*	*F*	*P*	*F*	*P*	*F*	*P*
Genotype	5.7	0.040	10.8	<0.001	5.3	0.042	1.5	NS
Environment	9.5	0.003	7.8	0.013	8.0	0.010	11.0	<0.001
Genotype × Environment	6.0	0.037	5.7	0.039	6.6	0.028	4.6	NS

### Soil physicochemical properties

**Figure 3:**
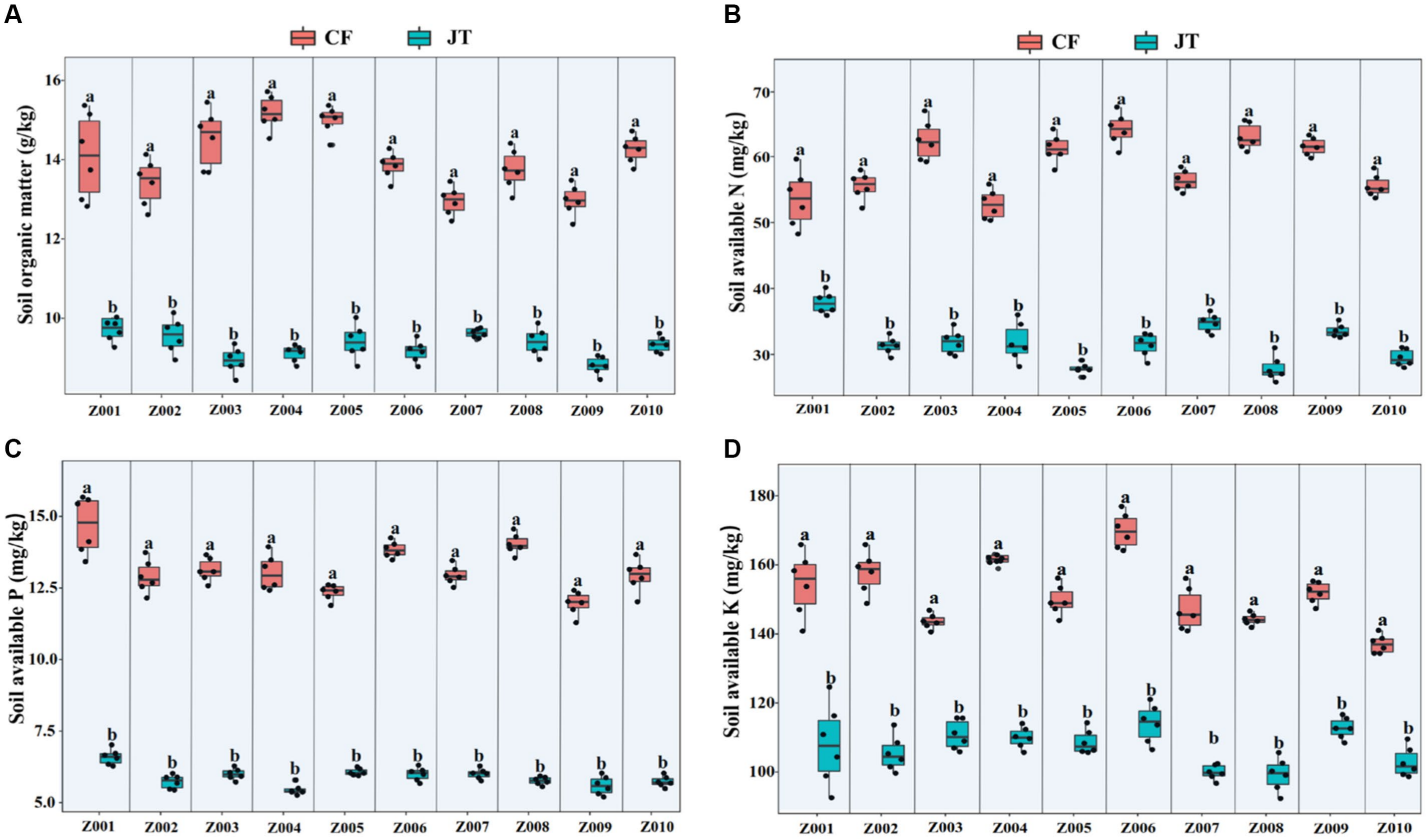
Rhizospheric soil physicochemical properties of different genotype licorice in different cultivation locations: **(A)** Soil organic matter, **(B)** Soil available N, **(C)** Soil available P, and **(D)** Soil available K. CF, Chifeng; JT, Jingtai. The different letters above the error line indicate significant difference at *p* < 0.05.

The effects of different genotype and environmental factors on the licorice rhizospheric soil nutrients were significantly different (Figure 3). On the whole, soil nutrient parameters in Chifeng were significantly higher than those in Jingtai. In terms of soil organic matter, genotypes Z004 and Z005 in Chifeng were significantly higher than other genotypes, while there was no significant difference between different genotypes in Jingtai (Figure 3A). In terms of soil available N content, the genotypes Z003, Z005, Z006, Z008, and Z009 in Chifeng had the highest content, while the Jingtai production areas had the highest concentrations of Z001, Z007, and Z009 (Figure 3B). In terms of soil available P content, the content of Z001, Z006, and Z008 were the highest in Chifeng, and there were no significant differences between different genotypes in Jingtai (Figure 3C). In terms of soil available K, the content of Z006 in Chifeng was the highest, and the content of Z010 was the lowest. In Jingtai, Z007 and Z008 had the lowest content (Figure 3D). Through two-way ANOVA, we found that genotype and environmental factors of different production areas had significant interaction effects on soil organic matter and available K content, while environmental factors had extremely significant effects on soil organic matter, available N, and available P (Table 5).

**Table 5:** Two-way ANOVA of the effect of genotype and environment condition on soil physiological parameter of licorice plants.

	Organic matter (g/kg)		Available N (mg/kg)		Available P (mg/kg)		Available K (mg/kg)	
	*F*	*P*	*F*	*P*	*F*	*P*	*F*	*P*
Genotype	1.2	NS	3.8	NS	2.3	NS	5.2	0.044
Environment	10.5	<0.001	9.8	0.001	9.2	0.008	7.5	0.011
Genotype × Environment	5.4	0.043	4.2	NS	4.3	NS	5.5	0.041

### Rhizospheric soil fungi community composition

**Figure 4:**
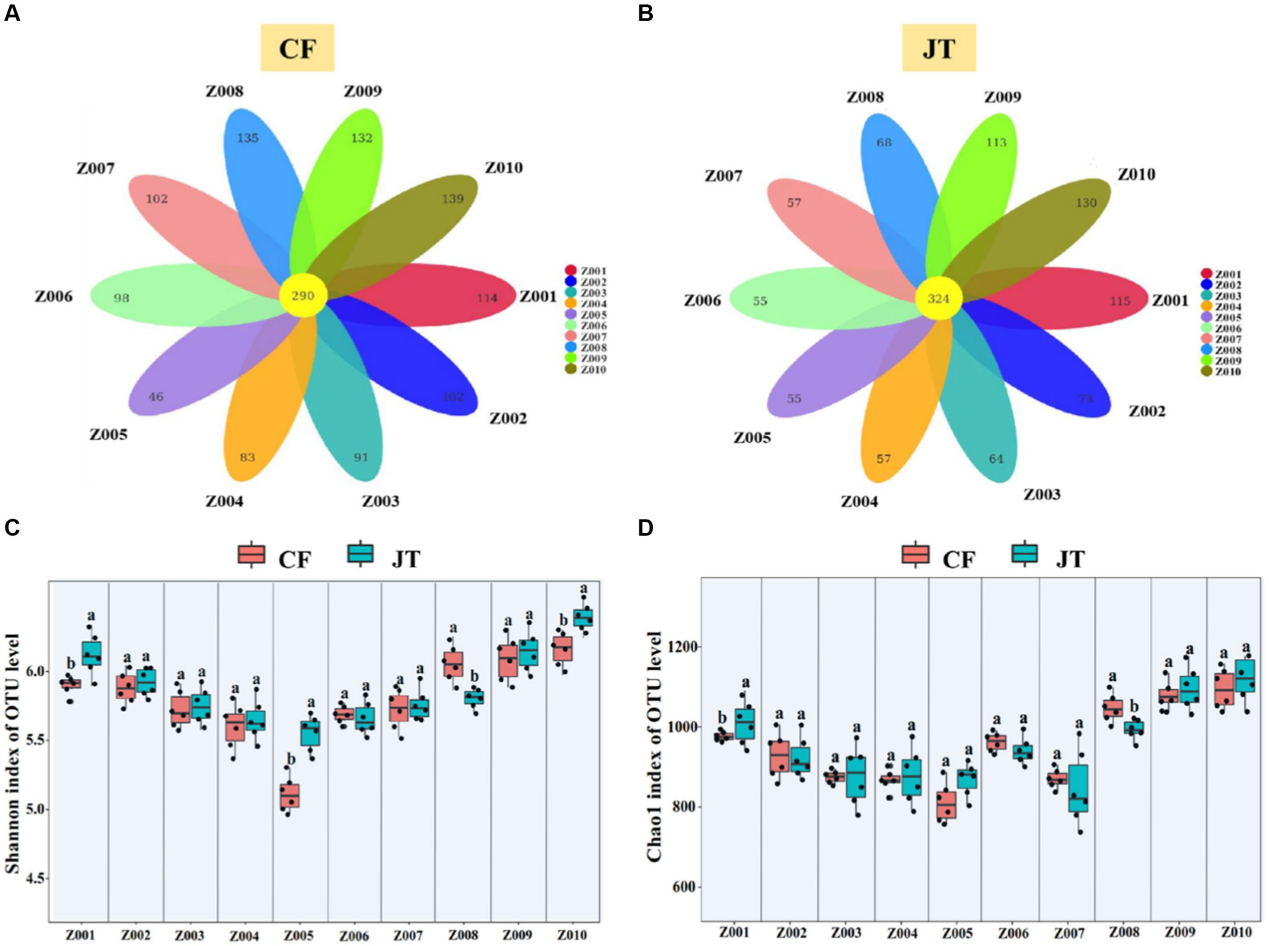
Rhizospheric soil fungal community composition of licorice genotype in different cultivation locations: **(A)** Differences in the composition of OTUs of rhizosphere fungi in different genotypes of licorice in Chifeng, **(B)** Differences in the composition of OTUs of rhizosphere fungi in different genotypes of licorice in Jingtai, **(C)** Shannon index of OUT level, and **(D)** Chao1 index of OUT level. The different letters above the error line indicate significant difference at *p* < 0.05.

A total of 2,188,210 fungal sequences in Chifeng and 2,067,075 fungal sequences in Jingtai were obtained. The sequencing results covered the biological information of most of the fungi in the soil samples. After filtering 1,305,281 and 1,289,995 low quality sequences, 882,929 and 777,080 available fungal sequences were clustered into 1,332 and 1,111 fungal operational taxonomic units (OTUs) at 97% sequence similarity. Of the 1,332 fungal OTUs in the soil of Chifeng, 290 occurred in all 10 genotypes, while 114, 102, 91, 83, 46, 98, 102, 135, 132, and 139 OTUs were found only in Z001-Z010, respectively (Figure 4A). Of the 1,111 fungal OTUs in the soil of Jingtai, 324 detected in all 10 genotypes 115, 73, 64, 57, 55, 55, 57, 68, 113, and 130 existed only in Z001-Z010, respectively (Figure 4B).

The Shannon index and Chao1 index of fungi in the different soil locations were significantly different. In Chifeng, the Shannon index in Z008, Z009, and Z010 were significantly higher than other genotypes, while Z005 had the lowest. In Jingtai, the Shannon index in Z010 was significantly higher than other genotypes (Figure 4C). In Chifeng, the Chao1index in Z009 and Z010 were significantly higher than other genotypes, while Z005 was significantly lower than in the other genotypes. In Jingtai, the Chao1 index in Z009 and Z010 were significantly higher than other genotypes (Figure 4D).

**Figure 5:**
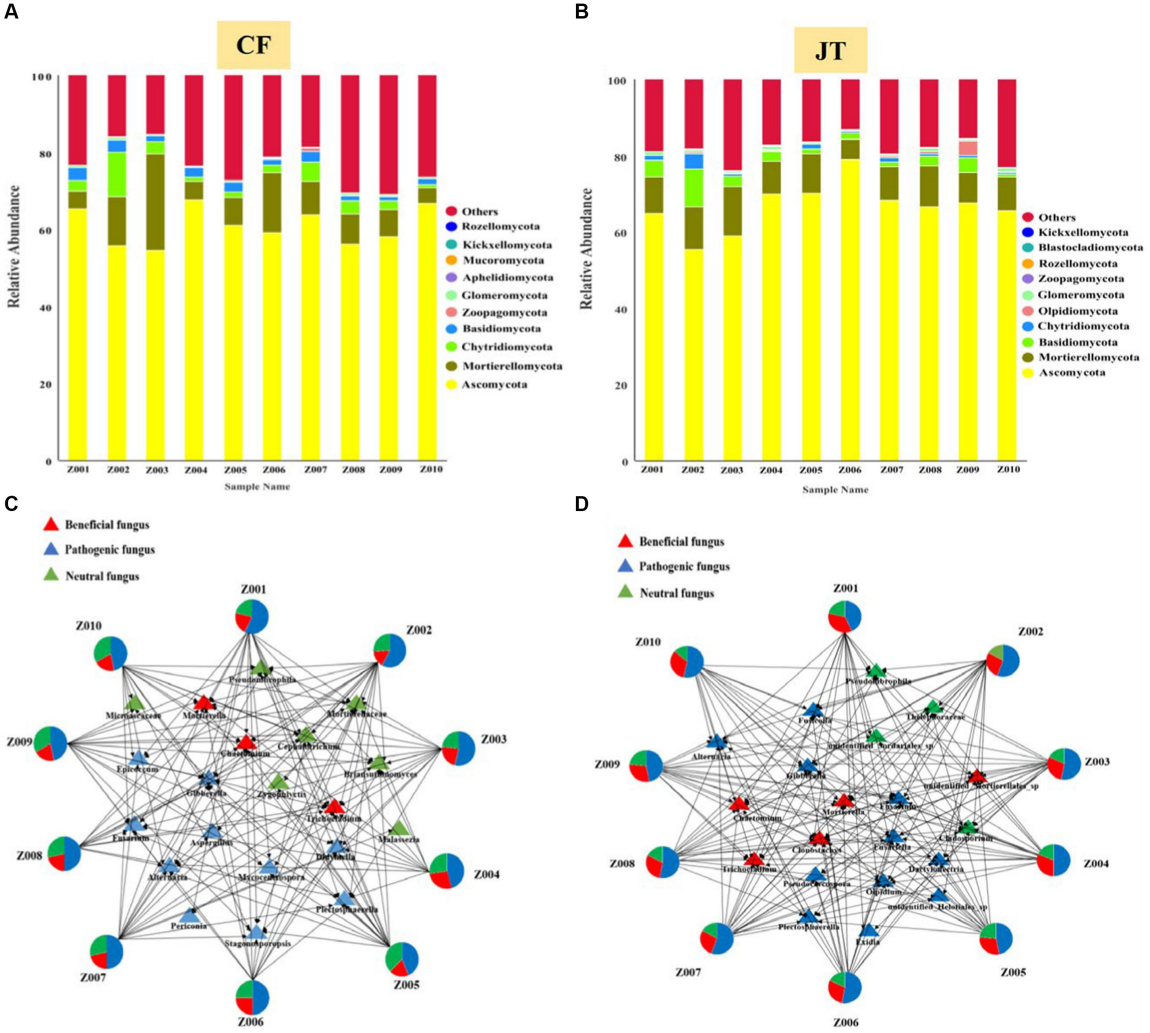
Relative abundance of rhizospheric soil fungal of different genotype and architecture of the genotype- rhizospheric soil fungal community network in different cultivation locations: **(A)** Relative abundance in Chifeng, **(B)** Relative abundance in Jingtai, Visualization network of beneficial, pathogenic and neutral rhizospheric soil fungal for licorice plants in Chifeng **(C)** and in Jingtai **(D)**.

A total of 1,332 fungal OTUs were found in the licorice rhizospheric of Chifeng, which were classified as *Ascomycota, Mortierellomycota, Chytridiomycota, Basidiomycota, Zoopagomycota, Glomeromycota, Aphelidiomycota, Mucoromycota, Kickxellomtcota, Rozellomycota* and certain unknown fungi. *Ascomycota, Mortierellomycota, Chytridiomycota, Basidiomycota*, and certain unknown fungi were identified in all treatments. *Ascomycota* was the dominant fungal phylum, with a relative abundance range of 54.5–67.6% across the various treatments. Interestingly, *Zoopagomycota* occurs only in the rhizospheric soils of the Z007 genotypes, while *Mucoromycota* was only not found in Z008 (Figure 5A). A total of 1,111 fungal OTUs were found in the licorice rhizospheric of Jingtai, which were classified as *Ascomycota, Mortierellomycota, Basidiomycota, Chytridiomycota, Olpidiomycota, Glomeromycota, Zoopagomycota, Rozellomycota, Blastocladiomucota, Kickxellomtcota*, and certain unknown fungi. *Ascomycota, Mortierellomycota, Basidiomycota*, and certain unknown fungi were identified in all treatments. *Ascomycota* was the dominant fungal phylum, with a relative abundance range of 55.4–78.9% across the various treatments. It is noteworthy that *Rozellomycota* only appeared in the rhizospheric of Z009 genotypes, while *Blastocladiomycota* did not colonize in the rhizospheric of Z001 or Z010 genotypes (Figure 5B). Furthermore, the variation in fungal community composition at the genus level under different sites were significant. The dominant fungal genera in rhizospheric soil of different genotypes licorice were also significantly different.

We screened mainly according to the abundance of rhizospheric soil fungal OUT and selected the top 20 genera with the highest abundance. The network structure analysis showed that the distribution of the top 20 fungal genera in the rhizospheric soil of different genotypes of licorice from different habitats was significantly different (Figures 5C, D). Specifically, in Chifeng, the fungal genera directly related to genotypes Z001, Z002, and Z007 were significantly more than other genotypes, while the fungal genera directly related to Z004 and Z008 were the least. According to the functional classification of strains, we found that the proportion of beneficial fungi in rhizospheric soil of licorice of the same genotype in Jingtai production area was significantly higher than that in Chifeng. In Chifeng, the beneficial fungi with genotype Z004 accounted for the largest proportion, reaching 27.1%. The beneficial fungi of Z002 accounted for the smallest proportion, only reaching 11.8%, while the pathogenic fungi were significantly higher than other genotypes, reaching 62.2%. In Jingtai, the fungal genera directly related to genotypes Z001 and Z009 were significantly more than other genotypes, and the fungal genera directly related to Z003 was the least. According to the functional classification of strains, we found that Z001 had the largest proportion of beneficial fungi, reaching 34.6%. The beneficial fungi of Z007 accounted for the smallest proportion, only 24.3%. Z002, Z007, Z008, and Z010 were the genotypes with a high proportion of pathogenic fungi, and there were no significant differences among the genotypes.

### Variation partitioning of growth parameters, active ingredient, and soil fungal community diversity

**Figure 6:**
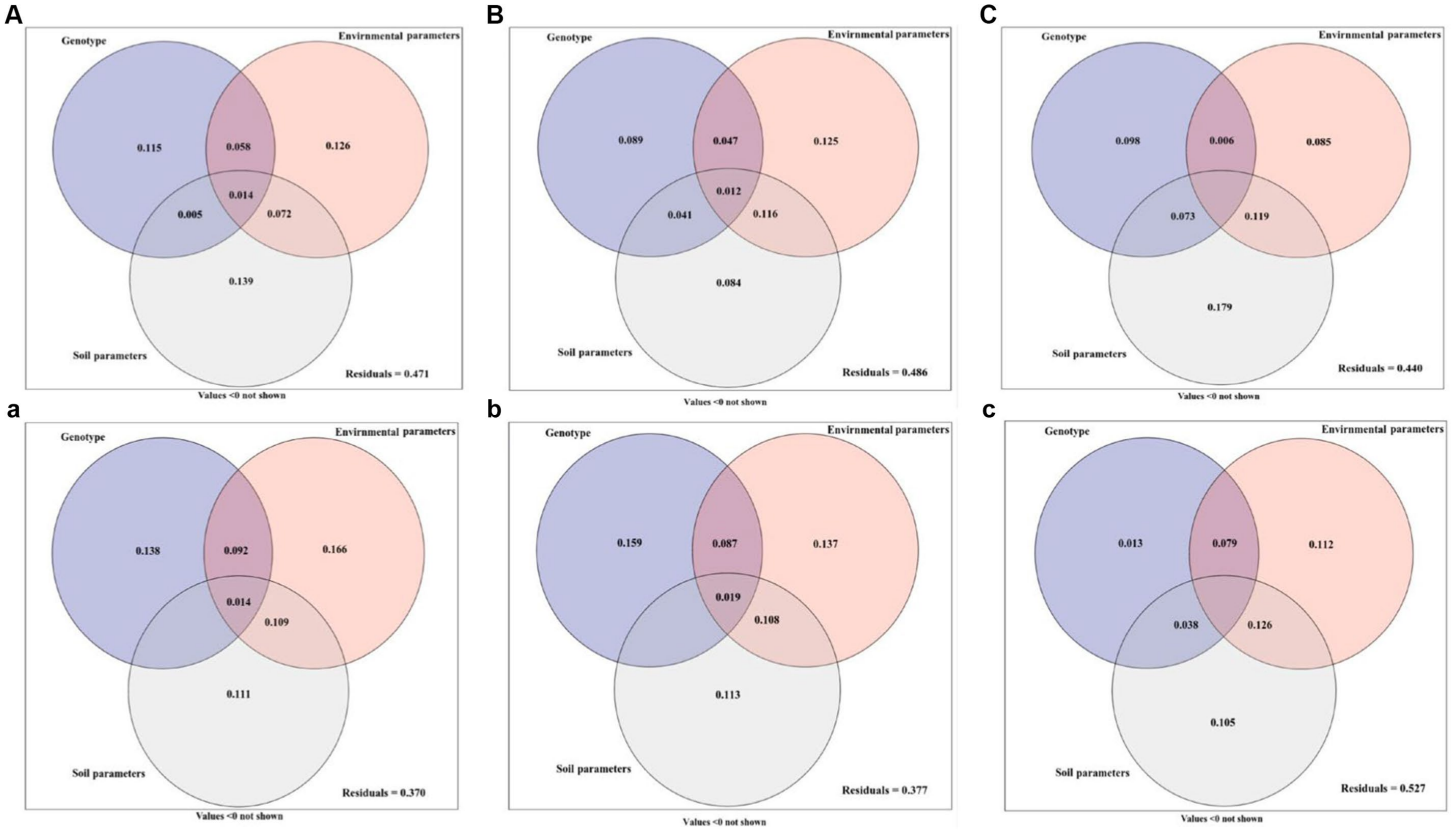
Variation partitioning of genotype, environment and soil parameter on the growth parameters **(A, a)**, active ingredient **(B, b)** and soil fungal community diversity **(C, c)** of licorice. (ABC: in Chifeng; abc: in Jingtai).

The growth parameters, active ingredients, and soil fungal community diversity of Licorice from different regions were quantitatively investigated by ANOVA as influenced by genotype, environment, and soil parameters (Figure 6). In Chifeng, the total explanation rate of genotype, environment, and soil parameters for plant growth parameters was 52.9%. Among these parameters, soil parameter was the most important factor affecting plant growth parameters, and the single explanation rate reached 13.9%. The explanation rates of genotype and environmental parameters were 11.5% and 12.6%, respectively (Figure 6A). A combination of genotype, environmental and soil parameters caused 51.4% diversification in the active ingredients. Among them, environmental parameters are the most important factors affecting the active components of plants, and the single explanation rate are 12.5%, and the explanation rates of genotype and soil parameters were 8.9% and 8.4%, respectively. It is interesting to note that the interaction between environmental and soil parameters explains 11.6% of the effect (Figure 6B). Genotype, environmental and soil parameters can explain 56.0% of the soil fungal community diversity. Among them, soil parameters were the dominant factors affecting the distribution of soil fungi, and the single explanation rate could reach 17.9%. The genotype and environmental parameters explained 9.8% and 8.5%. The interaction between environmental and soil parameters explained 11.9% of the effect, making it the second dominant factor after soil parameters (Figure 6C). In Jingtai, the total explanation rate of genotype and environmental and soil parameters for plant growth parameters was 63.0%. Among these parameters, environmental parameters were the most important factor affecting plant growth parameters, and the single explanation rate reached 16.6%. The explanation rates of genotype and soil parameter were 13.8% and 11.1%, respectively. The interaction between environmental and soil parameters explained 10.9% of the effect and was equally important in the system (Figure 6a). The content of active ingredients jointly explained by genotype and environmental and soil parameters for licorice was 62.3%, in which genotype was the primary factor affecting the accumulation of active ingredients, which alone explained 15.9%, and explanation rates of environmental and soil parameters were 13.7% and 11.3%, respectively (Figure 6b). Genotype and environmental and soil parameters could explain 47.3% of the soil fungal community diversity. Among these, environmental parameters and soil parameters were the dominant factors affecting the distribution of soil fungi, and the single explanation rate could reach 11.2% and10.5%, respectively. The genotype explained 1.3% and 8.5% of the effect. The interaction between environmental and soil parameters explained 12.6% of the effect, making it the second dominant factor after soil parameters (Figure 6c).

**Figure 7:**
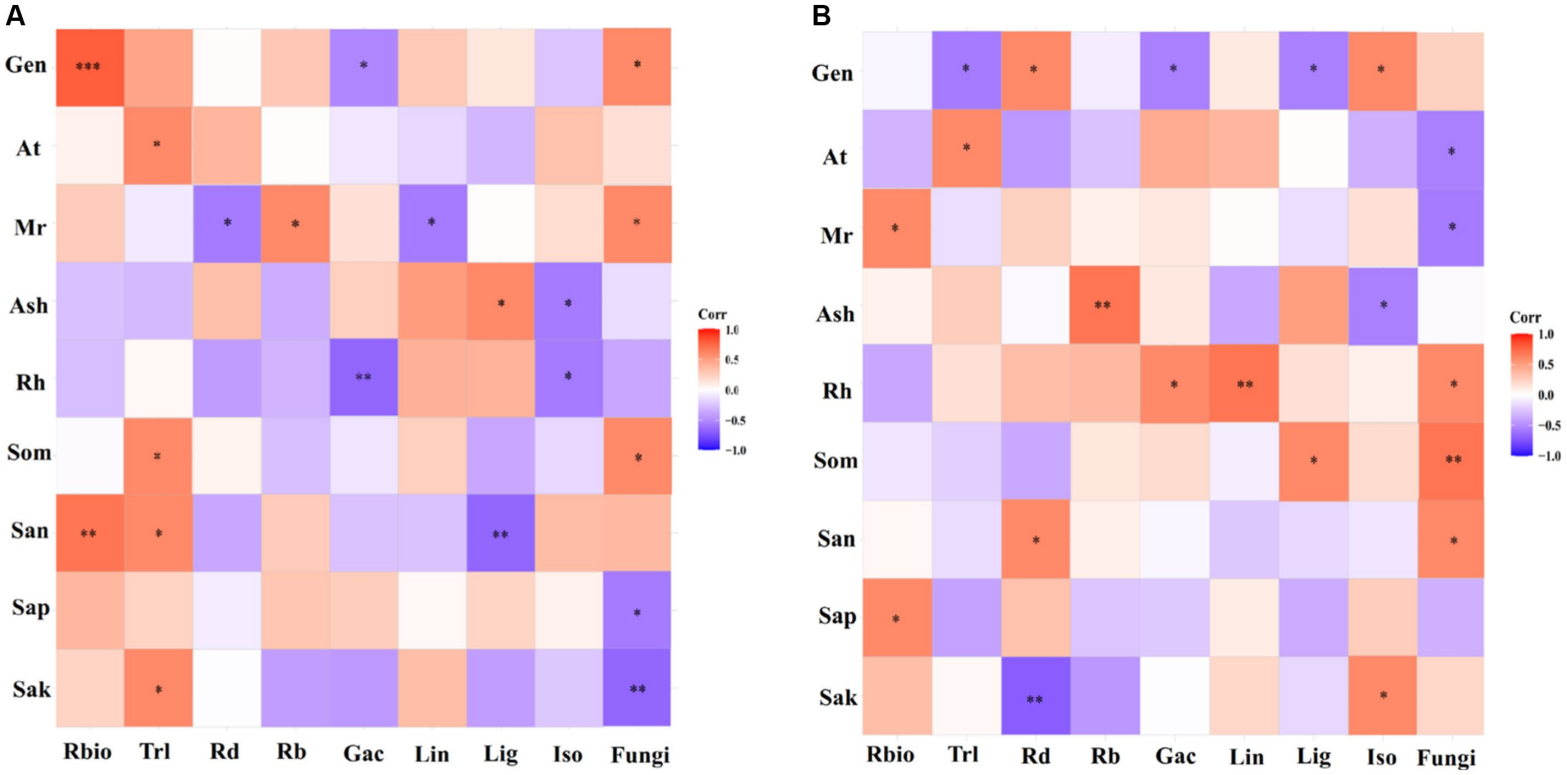
Preferences observed in the association between environmental factors-growth parameters-active ingredients-soil fungi in Chifeng **(A)** and Jingtai **(B)**. Gen, Genotype; At, Average temperature; Mr, Mean rainfall; Ash, Average sunshine; Rh, Relative humidity; Som, Soil organic matter; San, Soil available N; Sap, Soil available P; Sak, Soil available K; Rbio, Root biomass; Trl, Total root length; Rd, Root diameter; Rb, Root branch; Gac, Glycyrrhizic acid; Lin, Liquiritin; Lig, Liquiritigenin; Iso, Isoliquiritigenin.

Using correlation heat map analysis, we found that different degrees of preference were shown among genotype, environmental and soil parameters, and parameters of licorice growth, active ingredients, and rhizospheric fungal diversity in different production areas (Figure 7). In the Chifeng, root biomass was significantly and positively correlated with genotype and available soil N content. Total root length was significantly and positively correlated with mean temperature, soil organic matter, available N and K. Mean rainfall was significantly and positively correlated with the number of root branches, and it was significantly and negatively correlated with root diameter. Glycyrrhizic acid was negatively and significantly correlated with genotype and relative humidity. Liquiritin was negatively and significantly correlated with mean rainfall. Liquiritigenin was significantly and positively correlated with mean sunshine, while it was significantly and negatively correlated with available N content. Isoliquiritigenin was negatively and significantly correlated with mean sunshine and relative humidity. Soil fungal diversity was significantly and positively correlated with genotype, average rainfall, and soil organic matter, while it was significantly and negatively correlated with soil available P and K (Figure 7A). In Jingtai, root biomass was significantly and positively correlated with mean rainfall and soil available P. Total root length was significantly negatively correlated with genotype and positively correlated with mean air temperature. Root diameter was significantly positively correlated with genotype and available N, and negatively correlated with available K. The number of root branches was significantly and positively correlated with mean sunshine. Glycyrrhizic acid was negatively and significantly correlated with genotype and positively correlated with relative humidity. Liquiritin was significantly and positively correlated with relative humidity. Liquiritigenin was negatively and significantly correlated with genotype and positively with soil organic matter. Isoliquiritigenin was significantly and positively correlated with genotype and available K, while it was negatively correlated with mean sunshine. Rhizospheric fungal diversity was significantly and positively correlated with relative humidity, soil organic matter and available N, while it was significantly and negatively correlated with mean temperature and mean rainfall (Figure 7B).

### Correlation analyses

**Figure 8:**
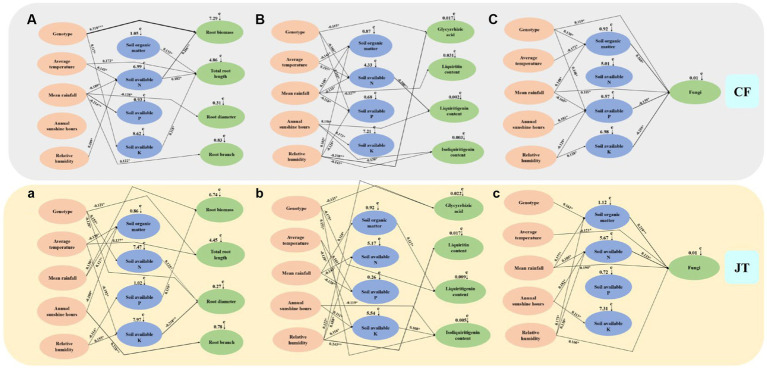
The causal relationships among genotype, environment, soil physicochemical properties, growth parameters, active ingredients and rhizospheric soil fungi community based on structural equation model (SEM). The final model fitted the data well: maximum likelihood, Growth characteristics of licorice in Chifeng: **(A)** *X*^2^ = 55.287, df = 9, *p* = 0.017, root mean square error of approximation = 0.239, goodness-of-fit index = 0.652, Akaike information criteria = 142.226; Active ingredients of licorice in Chifeng: **(B)** *X*^2^ = 61.187, df = 11, *p* = 0.009, root mean square error of approximation = 0.272, goodness-of-fit index = 0.627, Akaike information criteria = 150.634; Fungal abundance in the rhizospheric soil of licorice in Chifeng: **(C)** *X*^2^ = 53.780, df = 9, *p* = 0.019, root mean square error of approximation=0.226, goodness-of-fit index=0.671, Akaike information criteria=133.971;Growth characteristics of licorice in Jingtai: **(a)** *X*^2^ = 64.394, df = 11, *p* = 0.006, root mean square error of approximation=0.299, goodness-of-fit index=0.602, Akaike information criteria=158.925;Active ingredients of licorice in Jingtai: **(b)** *X*^2^ = 63.084, df = 11, *p* = 0.007, root mean square error of approximation=0.291, goodness-of-fit index=0.611, Akaike information criteria=155.441; **(c)** *X*^2^ = 58.192, df = 10, *p* = 0.013, root mean square error of approximation=0.258, goodness-of-fit index=0.636, Akaike information criteria=149.023. Solid lines and dashed lines indicate significant and non-significant pathways, respectively. The width of the solid lines indicates the strength of the causal effect, and the numbers near the arrows indicate the standardized path coefficients (^∗^*p* < 0.05, ^∗∗^*p* < 0.01, and ^∗∗∗^*p* < 0.001).

The mantel test and SEM models were used to construct model analysis of the main factors (genotype, environmental factors, and soil physicochemical properties) affecting the growth, active ingredient accumulation and rhizospheric soil fungal community diversity of licorice in two different cultivation locations, Chifeng and Jingtai (Figure 8). Among them, “+” indicate positive correlation, “–” indicate negative correlation, * indicate significant differences at *p* < 0.05, ** indicate significant differences at *p* < 0.01, and *** indicate significant differences at *p* < 0.001.

In terms of growth parameters (root biomass, total root length, root diameter, and number of root branches), the genotype of licorice had a very significant effect on root biomass in Chifeng, and the amount of the first level path was basically the same in the two production areas, the amount of the second level path in Chifeng was more than that in Jingtai production area. The effects of Chifeng model on growth parameters were mainly reflected in the following paths, among which the first level path: (1) Genotype $\overset{{+}^{***}}{\to }$ Root biomass, (2) Soil organic matter $\overset{{+}^{*}}{\to }$ Total root length, (3) Average temperature $\overset{{+}^{*}}{\to }$ Total root length, (4) Mean rainfall $\overset{{-}^{*}}{\to }$ Root diameter and (5) Mean rainfall $\overset{{+}^{*}}{\to }$ Root branch. The second level path: (1) Average temperature $\overset{{+}^{*}}{\to }$ Soil available N $\overset{{+}^{**}}{\to }$ Root biomass, (2) Mean rainfall $\overset{{-}^{*}}{\to }$ Soil available N $\overset{{+}^{**}}{\to }$ Root biomass, (3)Relative humidity $\overset{{+}^{*}}{\to }$ Soil available N $\overset{{+}^{**}}{\to }$ Root biomass, (4) Average temperature $\overset{{+}^{*}}{\to }$ Soil available N $\overset{{+}^{*}}{\to }$ Total root length, (5) Mean rainfall $\overset{{-}^{*}}{\to }$ Soil available N $\overset{{+}^{*}}{\to }$ Total root length, (6) Relative humidity $\overset{{+}^{*}}{\to }$ Soil available N $\overset{{+}^{*}}{\to }$ Total root length and (7)Genotype $\overset{{+}^{*}}{\to }$ Soil available K $\overset{{+}^{*}}{\to }$ Total root length. The impacts on growth parameters in the Jingtai model were mainly reflected in the following paths. The first level path: (1) Mean rainfall $\overset{{+}^{*}}{\to }$ Root biomass, (2) Genotype $\overset{{-}^{*}}{\to }$ Total root length, (3) Average temperature $\overset{{+}^{*}}{\to }$ Total root length, (4) Genotype $\overset{{-}^{*}}{\to }$ Root diameter and (5) Annual sunshine hours $\overset{{+}^{**}}{\to }$ Root branch. The second level path: (1) Relative humidity $\overset{{-}^{*}}{\to }$ Soil available P $\overset{{+}^{*}}{\to }$ Root biomass, (2) Genotype $\overset{{+}^{*}}{\to }$ Soil available N $\overset{{+}^{*}}{\to }$ Root diameter, (3) Average temperature $\overset{{-}^{*}}{\to }$ Soil available K $\overset{{-}^{**}}{\to }$ Root diameter and (4) Relative humidity $\overset{{+}^{*}}{\to }$ Soil available K $\overset{{-}^{**}}{\to }$ Root diameter.

In terms of active ingredient parameters (glycyrrhizic acid, liquiritin, liquiritigenin, and isoliquiritigenin), the effect of different production areas on the active ingredients of licorice was mainly through the first level path. The second level path in Chifeng was mainly through soil available N, and the second path in Jingtai was mainly through soil organic matter and available K. The impact of the Chifeng model on active ingredient parameters were mainly reflected in the following paths. The first level path: (1) Genotype $\overset{{-}^{*}}{\to }$ Gycyrrhizic acid, (2) Relative humidity $\overset{{-}^{*}}{\to }$ Glycyrrhizic acid, (3) Mean rainfall $\overset{{-}^{*}}{\to }$ Liquiritin, (4) Annual sunshine hours $\overset{{+}^{*}}{\to }$ Liquiritigenin, (5) Annual sunshine hours $\overset{{-}^{*}}{\to }$ Isoliquiritigenin and (6) Relative humidity $\overset{{-}^{*}}{\to }$ Isoliquiritigenin. The second level path: (1) Genotype $\overset{{-}^{*}}{\to }$ Soil available N $\overset{{-}^{**}}{\to }$ Liquiritigenin, (2) Average temperature $\overset{{+}^{*}}{\to }$ Soil available N $\overset{{-}^{**}}{\to }$ Liquiritigenin, (3) Mean rainfall $\overset{{-}^{*}}{\to }$ Soil available N $\overset{{-}^{**}}{\to }$ Liquiritigenin. The impact of Jingtai model on active ingredient parameters were mainly reflected in the following paths. The first level path: (1) Genotype $\overset{{-}^{*}}{\to }$ Gycyrrhizic acid and (2) Relative humidity $\overset{{+}^{*}}{\to }$ Glycyrrhizic acid, (3) Relative humidity $\overset{{+}^{**}}{\to }$ Liquiritin, (4) Genotype $\overset{{-}^{*}}{\to }$ Liquiritigenin, (5) Genotype $\overset{{+}^{*}}{\to }$ Isoliquiritigenin and (6) Annual sunshine hours $\overset{{-}^{*}}{\to }$ Isoliquiritigenin. The second level path: (1) Mean rainfall $\overset{{+}^{*}}{\to }$ Soil organic matter $\overset{{+}^{*}}{\to }$ Liquiritigenin, (2) Average temperature $\overset{{-}^{*}}{\to }$ Soil available K $\overset{{+}^{*}}{\to }$ Isoliquiritigenin, (3) Annual sunshine hours $\overset{{+}^{*}}{\to }$ Soil available K $\overset{{+}^{*}}{\to }$ Isoliquiritigenin and (4) Relative humidity $\overset{{+}^{*}}{\to }$ Soil available K $\overset{{+}^{*}}{\to }$ Isoliquiritigenin.

In terms of soil fungal diversity indices, the influence of different production areas on soil fungal diversity of licorice was mainly through the second level path. The second level path of Chifeng was mainly through soil organic matter, available P and K, and the second level path of Jingtai was mainly through soil organic matter and available N. The influence of Chifeng model were mainly reflected in the following ways. The first level path: (1) Genotype $\overset{{+}^{*}}{\to }$ Fungi and (2) Mean rainfall $\overset{{+}^{*}}{\to }$ Fungi. The second level path: (1) Genotype $\overset{{+}^{*}}{\to }$ Soil organic matter $\overset{{+}^{*}}{\to }$ Fungi, (2) Average temperature $\overset{{-}^{*}}{\to }$ Soil organic matter $\overset{{+}^{*}}{\to }$ Fungi, (3) Mean rainfall $\overset{{+}^{*}}{\to }$ Soil organic matter $\overset{{+}^{*}}{\to }$ Fungi, (4) Mean rainfall $\overset{{-}^{*}}{\to }$ Soil available P $\overset{{-}^{*}}{\to }$ Fungi, (5) Annual sunshine hours $\overset{{+}^{*}}{\to }$ Soil available P $\overset{{-}^{*}}{\to }$ Fungi, (6) Relative humidity $\overset{{-}^{*}}{\to }$ Soil available P $\overset{{-}^{*}}{\to }$ Fungi, (7) Relative humidity $\overset{{+}^{*}}{\to }$ Soil available K $\overset{{-}^{*}}{\to }$ Fungi. In terms of soil fungal diversity indices, the influence of Jingtai model were mainly reflected in the following ways, among which the first level path: (1) Average temperature $\overset{{-}^{*}}{\to }$ Fungi, (2) Mean rainfall $\overset{{+}^{*}}{\to }$ Fungi and (3) Relative humidity $\overset{{+}^{*}}{\to }$ Fungi. The second level path: (1) Genotype $\overset{{+}^{*}}{\to }$ Soil organic matter $\overset{{+}^{*}}{\to }$ Fungi, (2) Mean rainfall $\overset{{+}^{*}}{\to }$ Soil organic matter $\overset{{+}^{*}}{\to }$ Fungi, (3) Relative humidity $\overset{{+}^{*}}{\to }$ Soil organic matter $\overset{{+}^{*}}{\to }$ Fungi, (4) Mean rainfall $\overset{{+}^{*}}{\to }$ Soil available N $\overset{{+}^{*}}{\to }$ Fungi, (5) Annual sunshine hours $\overset{{+}^{*}}{\to }$ Soil available N $\overset{{+}^{*}}{\to }$ Fungi and (6) Relative humidity $\overset{{+}^{*}}{\to }$ Soil available N $\overset{{-}^{*}}{\to }$ Fungi.

## Discussion

### Effects of genotypes and environmental factors on growth characteristics and active ingredients of licorice

The current study has confirmed that there were large variations in the plant biomass and contents of active ingredients of 10 genotypes of licorice in two cultivation locations. The SEM analysis and variance partitioning showed that genotype and environmental factors were the main factors affecting growth parameters and active components, especially in the Chifeng production area where genotype had an extremely significant direct impact on root biomass. The growth and development of medicinal plants in natural habitats are mainly influenced by genetic and environmental factors, resulting in changes in phenotypic traits and accumulation of secondary metabolites (Leimar, 2009; Zhang J. et al., 2011; Wang and Zhou, 2017). Genetic factors, namely the variety characteristics of medicinal plants, determine the basic quality of this medicinal plant, which is an inherent attribute. The yield and quality of medicinal plants are directly related to genetic factors.

Our study found that licorice of the same genotype grew in Chifeng and Jingtai areas with different habitat conditions, and their growth traits and active ingredient parameters showed significant differences. For example, the root biomass of different licorice plants growing in Chifeng or Jingtai was significantly different. The root biomass of licorice genotype Z001 growing in Chifeng or Jingtai was significantly higher than that of others. It shows that the main reason of this difference at this time is due to the dominant factor of genotype. Meanwhile, the same genotype grown in Jingtai showed significantly higher root biomass parameters than those growing in Chifeng. It shows that the main reason of this difference at this time is due to the dominant factor of environment. A similar situation also occurred in terms of the indicators such as total root length, root diameter, and root branching. He et al., studied 6 different genotypes of *S. miltiorrhiza* from 5 producing areas and found that there were significant differences in plant biomass, active ingredients, rhizospheric soil physicochemical properties and microbial composition. From the perspective of comprehensive growth traits and medicinal components, DS993 was the most suitable genotype of *S. miltiorrhiza* from 5 producing areas (He et al., 2023). Studies have found that the *Cornus officinalis* tree species with ellipticform, cylindricform, and long pear-shaped fruit types are productive types, with good growth, strong resistance, and large fruits. These species all grew well in different production areas, indicating that they had good genetic stability (Chen et al., 2002). After years of natural selection, cultivation, and domestication, researchers have found that the newly cultivated *Scrophularia ningpoensis* Hemsl (Mixuanshen) has a great change in external morphology compared with *Scrophularia ningpoensis* Hemsl. (Maoxuanshen), and the Mixuanshen species have strong roots, which greatly improves the yield (Huang et al., 2004). Chen et al., comparing the growth and development status and biomass allocation of *Artemisia annua* from four different provenances, the results showed that the biomass of Du’an provenance was significantly higher than that of other provenances (Chen et al., 2008). In the study of different flowering stages of *Capsella rubella*, Niu et al., found that the flowering gene FLC had frequent transposon insertions in both the intergenic spacer and the gene region, where the gene structure was changed, and the expression was reduced, eventually leading to early flowering (Niu et al., 2019).

In addition, the glycyrrhizic acid content of the genotypes Z003 and Z004 grown in the two regions remained stable with no significant difference in active ingredient indicators. The glycyrrhizic acid content of other genotypes was significantly higher in Jingtai than in Chifeng. This indicates that different genotypes of the same plant also have different responses to the environment. A similar situation also occurred in terms of the indicators such as liquiritin and isoliquiritigenin content. The same genotype produces different phenotypic changes in response to different environmental conditions, which is considered to be the most important response feature of an organism to environmental conditions or stimuli (Huey and Gilchrist, 2000). The RAPD analysis of the genetic structure of *Atractylodes lancea* showed that for Mao-cangzhu genetic differentiation occurred in the process of long-term adaptation to the environment. This is consistent with the obvious difference between the volatile oil components of Mao-cangzhu and *A. lancea* of other production areas (Guo et al., 2006). Zhou et al., reported the specific expression patterns of candidate genes related to anthraquinone biosynthesis in different tissues in *Rheum tanguticum* (Zhou et al., 2021). It was found that the contents of liquiritin, glycyrrhizic acid, flavonoids, and polysaccharides in different variant types of *G. uralensis* Fisch vary greatly, indicating that the variation of *G. uralensis* Fisch phenotype has caused changes in the chemical composition of the medicinal material (Yang et al., 2007), which is similar to the results of this study. Cai et al., found that there were significant differences in total phenols, flavonoids, and antioxidant activities in *Houttuynia cordata* from different provenances (Cai et al., 2013). These results indicated that there was an inseparable relationship between genetic background, environmental factors, and their chemical composition and content.

### Effects of genotypes and environmental factors on physicochemical properties and fungal communities in rhizospheric soil of licorice

Soil physicochemical properties are important indicators for evaluating soil quality. Rhizospheric is the most important medium connecting plants and soil, it is also the central area for the interaction of plants, soil, microorganisms, and their environment. The changes of rhizospheric microbial community structure and diversity can intuitively reflect the dynamic changes of soil quality and nutrients in the rhizospheric. As an important microorganism, fungi play a vital role in improving soil structure and materials and the energy cycle in the soil system. Therefore, it is of great significance to study the diversity of soil fungi and its relationship with soil physical and chemical properties (He et al., 2022). The SEM analysis and variance partitioning showed that soil physicochemical properties of licorice rhizospheric soil were more affected by environmental factors than genotypes, and soil physicochemical property parameters were the main factors affecting soil fungal diversity. For example, by analyzing the effects of different production regions and environmental conditions on the soil physiological parameters of licorice, it was found that the environmental factors in Chifeng and Jingtai had significant differences in the four indices of soil physicochemical properties. There were significant interactions between production regions and environmental factors on the contents of organic matter and available K. Correspondingly, the soil physicochemical properties of the two production areas were the main factors that directly affected the growth and the content of the active ingredient of licorice, but the effects of different soil factors were also significantly different. For example, in Chifeng, soil available N has a positive impact on root biomass and total root length but has a negative impact on the content of liquiritigenin. In Jingtai production area, soil available N increased the root diameter, soil organic matter increased the content of liquiritigenin, and soil available P increased the content of isoliquiritigenin, but decreased the root diameter. The ecological environment has an important impact on the quality of medicinal plants. In recent years, many scholars have carried out research on soil environments and adaptive production areas. For example, Chen et al., revealed the characteristics of the soil environment in the authentic production areas of Sichuan genuine medicinal materials, such as *Rhizoma Coptidis*, *Angelica dahuricae*, *Ophiopogon japonicus*, and *Lonicera japonica* (Chen et al., 1996a,b, 2003; Ding et al., 1996). By studying the key soil factors for the accumulation of active components in *Scutellaria baicalin*, Jiang found that the content of baicalin was positively correlated with soil pH, and negatively correlated with organic matter. Dihydroflavones were positively correlated with soil pH, and negatively correlated with ammonium nitrogen, potassium ion, and organic matter (Jiang, 2021). Lin et al., through the investigation and study of soil ecological factors and meteorological factors in licorice production areas, believed that climate factors were the prerequisite for the survival of licorice (Lin and Lin, 1992), while soil factors affected the quality of licorice, which was similar to the results of our study.

Soil microorganisms are sensitive to environmental factor change, and soil physicochemical properties are the main factors affecting the diversity of soil fungi. In terms of soil fungal diversity indicators, the soil parameters in Chifeng are the dominant factors affecting the distribution of soil fungi. The soil fungal diversity is significantly positively correlated with soil organic matter, genotype, and average rainfall, and significantly negatively correlated with soil available P and K. In Jingtai, environmental parameters and soil parameters are the main factors affecting the distribution of soil fungi. The diversity of soil fungi is significantly positively correlated with relative humidity, soil organic matter and available N, and significantly negatively correlated with average temperature and average rainfall. In addition, the rhizospheric soil fungal community of 10 different genotypes licorice in both production areas include *Ascomycota, Mortierellomycota* and *Basidiomycota*, which to some extent indicates that they are common fungal community of licorice. The *Aphelidiomycota* and *Mucoromycota* exclusively occur in licorice plants found in the Chifeng region, whereas the *Blastocladiomucota* and *Olpidiomycota* are only present in licorice plants in the Jingtai region. There are also distinct fungal community observed in the two production areas, suggesting that environmental factors play a significant role in their composition to some extent. Meanwhile, rhizospheric soil fungal genera Malassezia, Periconia and Epicoccum exclusively occur in the Z001 genotype of licorice growing in the Chifeng region, while fungal genera Exidia only appears in the Z001 and Z003 genotypes of licorice growing in the Jingtai region. To some extent, it indicates that the reason for this difference fungal genera under the same environment is dominated by genotype. In recent years, the potential application of increasing soil fungal biodiversity to improve soil quality and ecosystem productivity has become a new hot spot in soil research. Liu studied the effects of poplar genotypes and mixed modes on rhizospheric soil nutrients and microbiological characteristics and found that different poplar genotypes had significant differences in rhizospheric soil nutrients and rhizospheric soil bacterial and fungal communities in different seasons (Liu, 2014). He et al., studied the effects of dark septate endophytes (DSE) on the performance and rhizospheric soil microbial composition of *Lycium ruthenicum* Murr under drought stress. They found that soil microbial communities also showed significant changes after inoculation with DSE under different water conditions (He et al., 2022). Guan found that N-efficient cucumber varieties can improve their nitrogen adsorption and utilization ability by regulating their rhizospheric soil enzyme activity, microbial number, and microbial community structure (Guan, 2013). Meng found that the main groups of soil fungi varied from season to season, and the number of soil fungi was significantly correlated with temperature and rainfall (Meng, 2007). Guo studied the impact mechanism of rainfall pattern changes on soil microbial community and ecological multifunctionality and found that rainfall indirectly affected ecological multifunctionality by changing soil nutrients and fungal community composition (Guo, 2022). Research showed that soil fungal communities were greatly affected by environmental factors, such as different vegetation types and different litter types, which led to significant differences in soil physicochemical properties, such as soil pH, moisture content, biomass, and chemical properties of litter, and underground carbon distribution. Yang et al., found that the composition of soil fungal community was significantly related to plant diversity, soil pH, annual average temperature, soil organic matter, annual average rainfall, soil moisture content and altitude. Among these, plant diversity is the main factor affecting fungal diversity (Yang et al., 2022).

## Conclusion

This study showed that when 10 different genotypes of licorice were planted in two different growth environments, their growth characteristics, active ingredient content, rhizospheric soil physicochemical properties, and rhizospheric soil fungal community would show certain differences. Through SEM models and variance partitioning, it was shown that there were also certain differences in the number and intensity of pathways in different cultivation areas. Both genotype and environmental factors directly or indirectly affect the growth traits and active ingredient content of licorice. Among these, genotype was the main factor affecting the growth traits of licorice, while environmental factors and soil physicochemical properties were the main factors affecting the active ingredients of licorice. In addition, the rhizospheric soil fungal community was significantly affected by soil physicochemical properties. The same genotype of licorice in different cultivation areas or different genotypes of licorice in the same cultivation area produced distinct fungal community. Therefore, we suggest that changing the composition of soil fungal community may be valuable for optimizing the cultivation of licorice. The obtained data showed that there was a complex relationship between genotype, environmental factors, soil characteristics, plant growth, and active ingredient content, but it should be noted that selecting the appropriate genotype and cultivation site was essential to maximize the yield and quality of licorice. These findings are helpful to understand the ecological adaptability of different genotypes of licorice resources, and to select appropriate licorice genotypes for specific planting areas, so as to maximize yield and quality. To sum up, according to the growth characteristics and active components, Z009 is the most suitable genotype to plant in Jingtai. From the perspective of active ingredients, Z010 is the most suitable genotype for licorice cultivation in the two production areas.

## Data availability statement

The datasets presented in this study can be found in online repositories. The names of the repository/repositories and accession number(s) can be found at: The Illumina MiSeq sequence datasets are available at the NCBI Sequence Read Archive BioProject ID PRJNA1016103 (SUB13828680).

## Author contributions

TH: Conceptualization, Data curation, Formal analysis, Methodology, Resources, Writing – original draft, Writing – review & editing. XL: Conceptualization, Formal analysis, Funding acquisition, Investigation, Project administration, Resources, Writing – review & editing. DL: Data curation, Formal analysis, Methodology, Visualization, Writing – review & editing. CJ: Data curation, Formal analysis, Methodology, Visualization, Writing – review & editing. CC: Conceptualization, Funding acquisition, Investigation, Project administration, Resources, Writing – review & editing. CH: Conceptualization, Data curation, Formal analysis, Investigation, Methodology, Project administration, Resources, Software, Visualization, Writing – original draft, Writing – review & editing.

## Footnotes

^1^ http://fungene.cme.msu.edu/

^2^ http://www.stbates.org/guilds/app.php
