# Supplementary material for: Genotype and environment factors driven licorice growth and rhizospheric soil fungal community changes
Source: Front Microbiol. 2023 Nov 23;14:1308412. doi: 10.3389/fmicb.2023.1308412 (PMC10701759; doi:10.3389/fmicb.2023.1308412)
Supplement: Supplementary file 1 [file Data_Sheet_1.DOCX]

**Table S1** HPLC determination mobile phase parameters of active ingredients in Licorice.

| Time (min) | A (Acetonitrile %) | B (0.5 % Phosphate aqueous solution %) |
| --- | --- | --- |
| 0～8 | 20 | 80 |
| 8～30 | 20→38 | 80→62 |
| 30～42 | 38→50 | 62→50 |
| 42～45 | 50→95 | 50→5 |
| 45～50 | 95→20 | 5→80 |

**Table S2** Mantel tests showing correlationships (R values) between genotype, climatic, soil physicochemical parameters and growth parameters in Chifeng.

| Variable | Gen | At | Mr | Ash | Rh | Som | San | Sap | Sak | Rbio | Trl | Rd | Rb |
| --- | --- | --- | --- | --- | --- | --- | --- | --- | --- | --- | --- | --- | --- |
| Gen | 1 |  |  |  |  |  |  |  |  |  |  |  |  |
| At | 0.084 | 1 |  |  |  |  |  |  |  |  |  |  |  |
| Mr | 0.021 | -0.217** | 1 |  |  |  |  |  |  |  |  |  |  |
| Ash | -0.062 | 0.164* | -0.072 | 1 |  |  |  |  |  |  |  |  |  |
| Rh | -0.127* | -0.117* | 0.255** | -0.118* | 1 |  |  |  |  |  |  |  |  |
| Som | 0.059 | 0.052 | 0.086 | -0.072 | 0.089 | 1 |  |  |  |  |  |  |  |
| San | -0.077 | 0.138* | -0.149* | 0.039 | 0.113* | 0.106* | 1 |  |  |  |  |  |  |
| Sap | 0.018 | -0.058 | -0.237** | -0.056 | -0.075 | -0.011 | -0.067 | 1 |  |  |  |  |  |
| Sak | 0.132* | 0.119* | -0.058 | 0.033 | -0.036 | 0.090 | 0.019 | -0.067 | 1 |  |  |  |  |
| Rbio | 0.307*** | 0.095 | 0.085 | 0.098 | 0.023 | 0.066 | 0.289** | 0.050 | 0.071 | 1 |  |  |  |
| Trl | 0.093 | 0.187* | -0.029 | 0.067 | 0.077 | 0.146* | 0.197* | -0.072 | 0.126* | 0.282** | 1 |  |  |
| Rd | 0.066 | 0.071 | -0.113* | 0.051 | -0.026 | 0.099 | 0.082 | -0.016 | -0.022 | 0.163* | -0.051 | 1 |  |
| Rb | -0.049 | -0.060 | 0.134* | -0.094 | -0.081 | -0.007 | 0.037 | 0.028 | 0.083 | 0.076 | 0.188* | -0.106* | 1 |

Note: The significance identifiers in the table mean *P<0.05, **P<0.01, and ***P<0.001. Pr, provenance; At, average temperature; Mr, mean rainfall; Ash, annual sunshine hours; Rh, relative humidity; Som, soil organic matter; San, soil available N; Sap, soil available P; Sak, soil available K; Rbio, root biomass; Trl, total root length; Rd, root diameter; Rb, root branch.

**Table S3** Mantel tests showing correlationships (R values) between genotype, climatic, soil physicochemical parameters and active ingredients in Chifeng.

| Variable | Gen | At | Mr | Ash | Rh | Som | San | Sap | Sak | Gac | Lin | Lig | Iso |
| --- | --- | --- | --- | --- | --- | --- | --- | --- | --- | --- | --- | --- | --- |
| Gen | 1 |  |  |  |  |  |  |  |  |  |  |  |  |
| At | 0.072 | 1 |  |  |  |  |  |  |  |  |  |  |  |
| Mr | 0.058 | -0.183* | 1 |  |  |  |  |  |  |  |  |  |  |
| Ash | -0.016 | 0.127* | -0.058 | 1 |  |  |  |  |  |  |  |  |  |
| Rh | -0.087 | -0.092 | 0.204** | -0.140* | 1 |  |  |  |  |  |  |  |  |
| Som | 0.034 | -0.125* | 0.132* | -0.051 | 0.149* | 1 |  |  |  |  |  |  |  |
| San | -0.179* | 0.112* | -0.115* | -0.069 | 0.034 | 0.098 | 1 |  |  |  |  |  |  |
| Sap | 0.114* | -0.036 | -0.129* | 0.018 | -0.114* | -0.042 | -0.085 | 1 |  |  |  |  |  |
| Sak | 0.069 | 0.097 | -0.034 | 0.182* | -0.083 | 0.073 | 0.004 | -0.079 | 1 |  |  |  |  |
| Gac | -0.103* | 0.062 | -0.071 | 0.094 | -0.188* | -0.066 | 0.057 | 0.019 | 0.097 | 1 |  |  |  |
| Lin | -0.042 | -0.010 | -0.109* | 0.036 | -0.072 | -0.007 | 0.032 | 0.008 | 0.062 | 0.105* | 1 |  |  |
| Lig | 0.029 | 0.041 | 0.038 | 0.173* | 0.033 | 0.046 | -0.231** | -0.015 | 0.005 | 0.089 | 0.066 | 1 |  |
| Iso | 0.060 | 0.057 | 0.082 | -0.122* | -0.151* | 0.028 | -0.088 | -0.084 | -0.027 | -0.012 | 0.031 | -0.059 | 1 |

Note: The significance identifiers in the table mean *P<0.05 and **P<0.01. Pr, provenance; At, average temperature; Mr, mean rainfall; Ash, annual sunshine hours; Rh, relative humidity; Som, soil organic matter; San, soil available N; Sap, soil available P; Sak, soil available K; Gac, glycyrrhizic acid; Lin, liquiritin; Lig, liquiritigenin; Iso, isoliquiritigenin.

**Table S4** Mantel tests showing correlationships (R values) between genotype, climatic, soil physicochemical parameters and fungi diversity in Chifeng.

| Variable | Gen | At | Mr | Ash | Rh | Som | San | Sap | Sak | Fungi |
| --- | --- | --- | --- | --- | --- | --- | --- | --- | --- | --- |
| Gen | 1 |  |  |  |  |  |  |  |  |  |
| At | 0.041 | 1 |  |  |  |  |  |  |  |  |
| Mr | 0.079 | -0.124* | 1 |  |  |  |  |  |  |  |
| Ash | -0.003 | 0.159* | -0.081 | 1 |  |  |  |  |  |  |
| Rh | -0.116* | -0.046 | 0.172** | -0.109* | 1 |  |  |  |  |  |
| Som | 0.121* | -0.186* | 0.119* | 0.034 | 0.055 | 1 |  |  |  |  |
| San | 0.052 | 0.091 | 0.015 | 0.008 | 0.073 | 0.081 | 1 |  |  |  |
| Sap | 0.163* | -0.055 | -0.138* | 0.126* | -0.142* | -0.050 | -0.062 | 1 |  |  |
| Sak | -0.076 | 0.072 | -0.066 | 0.082 | 0.136* | 0.089 | 0.015 | -0.054 | 1 |  |
| Fungi | 0.130* | -0.061 | 0.117* | -0.071 | 0.091 | 0.182* | 0.096 | -0.147* | -0.274** | 1 |

Note: The significance identifiers in the table mean *P<0.05 and **P<0.01. Pr, provenance; At, average temperature; Mr, mean rainfall; Ash, annual sunshine hours; Rh, relative humidity; Som, soil organic matter; San, soil available N; Sap, soil available P; Sak, soil available K.

**Table S5** Mantel tests showing correlationships (R values) between genotype, climatic, soil physicochemical parameters and growth parameters in Jingtai.

| Variable | Gen | At | Mr | Ash | Rh | Som | San | Sap | Sak | Rbio | Trl | Rd | Rb |
| --- | --- | --- | --- | --- | --- | --- | --- | --- | --- | --- | --- | --- | --- |
| Gen | 1 |  |  |  |  |  |  |  |  |  |  |  |  |
| At | 0.089 | 1 |  |  |  |  |  |  |  |  |  |  |  |
| Mr | 0.042 | -0.116* | 1 |  |  |  |  |  |  |  |  |  |  |
| Ash | -0.028 | 0.131* | -0.117* | 1 |  |  |  |  |  |  |  |  |  |
| Rh | 0.073 | -0.105* | 0.164* | -0.091 | 1 |  |  |  |  |  |  |  |  |
| Som | 0.095 | -0.114* | 0.113* | -0.154* | 0.066 | 1 |  |  |  |  |  |  |  |
| San | 0.172* | 0.077 | -0.019 | -0.011 | -0.003 | 0.048 | 1 |  |  |  |  |  |  |
| Sap | 0.059 | -0.024 | -0.073 | -0.052 | -0.123* | 0.001 | -0.018 | 1 |  |  |  |  |  |
| Sak | -0.007 | -0.176* | 0.028 | -0.063 | 0.118* | -0.035 | 0.033 | -0.079 | 1 |  |  |  |  |
| Rbio | 0.084 | 0.042 | 0.129* | 0.077 | 0.085 | 0.080 | 0.057 | 0.102* | 0.055 | 1 |  |  |  |
| Trl | -0.107* | 0.125* | 0.072 | 0.032 | 0.059 | 0.031 | 0.011 | -0.015 | 0.071 | 0.215** | 1 |  |  |
| Rd | -0.113* | -0.007 | 0.054 | 0.029 | -0.022 | 0.099 | 0.138* | 0.037 | -0.223** | 0.107* | -0.067 | 1 |  |
| Rb | 0.046 | -0.053 | -0.039 | 0.203** | -0.090 | -0.003 | -0.020 | -0.069 | 0.083 | 0.182* | 0.191* | -0.112* | 1 |

Note: The significance identifiers in the table mean *P<0.05 and **P<0.01. Pr, provenance; At, average temperature; Mr, mean rainfall; Ash, annual sunshine hours; Rh, relative humidity; Som, soil organic matter; San, soil available N; Sap, soil available P; Sak, soil available K; Rbio, root biomass; Trl, total root length; Rd, root diameter; Rb, root branch.

**Table S6** Mantel tests showing correlationships (R values) between genotype, climatic, soil physicochemical parameters and active ingredients in Jingtai.

| Variable | Gen | At | Mr | Ash | Rh | Som | San | Sap | Sak | Gac | Lin | Lig | Iso |
| --- | --- | --- | --- | --- | --- | --- | --- | --- | --- | --- | --- | --- | --- |
| Gen | 1 |  |  |  |  |  |  |  |  |  |  |  |  |
| At | 0.073 | 1 |  |  |  |  |  |  |  |  |  |  |  |
| Mr | 0.051 | -0.102* | 1 |  |  |  |  |  |  |  |  |  |  |
| Ash | -0.016 | 0.119* | -0.145* | 1 |  |  |  |  |  |  |  |  |  |
| Rh | 0.059 | -0.087 | 0.193* | -0.079 | 1 |  |  |  |  |  |  |  |  |
| Som | 0.082 | -0.055 | 0.126* | -0.090 | 0.045 | 1 |  |  |  |  |  |  |  |
| San | 0.158* | 0.034 | -0.118* | -0.005 | 0.172* | 0.021 | 1 |  |  |  |  |  |  |
| Sap | 0.067 | -0.006 | -0.107* | -0.032 | -0.029 | 0.013 | -0.031 | 1 |  |  |  |  |  |
| Sak | -0.015 | -0.113* | 0.063 | 0.101* | 0.130* | -0.008 | 0.070 | -0.069 | 1 |  |  |  |  |
| Gac | -0.137* | 0.028 | -0.089 | 0.084 | 0.128* | 0.057 | -0.012 | 0.038 | 0.050 | 1 |  |  |  |
| Lin | -0.076 | 0.002 | -0.053 | 0.009 | 0.279** | 0.012 | -0.054 | 0.075 | 0.016 | 0.071 | 1 |  |  |
| Lig | -0.142* | -0.061 | 0.006 | -0.017 | 0.076 | 0.124* | -0.005 | -0.018 | 0.089 | -0.008 | 0.006 | 1 |  |
| Iso | 0.163* | -0.015 | 0.047 | -0.136* | 0.041 | 0.088 | -0.027 | -0.082 | 0.113* | 0.026 | 0.059 | -0.020 | 1 |

Note: The significance identifiers in the table mean *P<0.05 and **P<0.01. Pr, provenance; At, average temperature; Mr, mean rainfall; Ash, annual sunshine hours; Rh, relative humidity; Som, soil organic matter; San, soil available N; Sap, soil available P; Sak, soil available K; Gac, glycyrrhizic acid; Lin, liquiritin; Lig, liquiritigenin; Iso, isoliquiritigenin.

**Table S7** Mantel tests showing correlationships (R values) between genotype, climatic, soil physicochemical parameters and fungi diversity in Jingtai.

| Variable | Gen | At | Mr | Ash | Rh | Som | San | Sap | Sak | Fungi |
| --- | --- | --- | --- | --- | --- | --- | --- | --- | --- | --- |
| Gen | 1 |  |  |  |  |  |  |  |  |  |
| At | 0.049 | 1 |  |  |  |  |  |  |  |  |
| Mr | 0.032 | -0.076 | 1 |  |  |  |  |  |  |  |
| Ash | -0.026 | 0.135* | -0.109* | 1 |  |  |  |  |  |  |
| Rh | 0.041 | -0.053 | 0.150* | -0.101* | 1 |  |  |  |  |  |
| Som | 0.127* | -0.021 | 0.162* | -0.078 | 0.186* | 1 |  |  |  |  |
| San | 0.075 | 0.068 | 0.117* | 0.164* | 0.152* | 0.047 | 1 |  |  |  |
| Sap | 0.050 | -0.013 | -0.078 | -0.016 | -0.004 | 0.030 | -0.010 | 1 |  |  |
| Sak | -0.048 | -0.088 | 0.052 | 0.120* | 0.089 | -0.021 | 0.043 | -0.057 | 1 |  |
| Fungi | 0.081 | -0.108* | 0.202**. | -0.035 | 0.175* | 0.251** | 0.146* | 0.038 | -0.009 | 1 |

Note: The significance identifiers in the table mean *P<0.05, **P<0.01, and ***P<0.001. Pr, provenance; At, average temperature; Mr, mean rainfall; Ash, annual sunshine hours; Rh, relative humidity; Som, soil organic matter; San, soil available N; Sap, soil available P; Sak, soil available K.
